# Norepinephrine and Vasopressin in Hemorrhagic Shock: A Focus on Renal Hemodynamics

**DOI:** 10.3390/ijms24044103

**Published:** 2023-02-17

**Authors:** Nicolas Fage, Pierre Asfar, Peter Radermacher, Julien Demiselle

**Affiliations:** 1 Department of Medical Intensive Care, University Hospital of Angers, 49100 Angers, France; 2 MITOVASC Laboratory, UMR INSERM (French National Institute of Health and Medical Research), 1083–CNRS 6015, University of Angers, 49933 Angers, France; 3 Institut Für Anästhesiologische Pathophysiologie und Verfahrensentwicklung, Universitätsklinikum, Helmholtzstrasse 8-1, 89081 Ulm, Germany; 4 Department of Intensive Care (Service de Médecine Intensive–Réanimation), Nouvel Hôpital Civil, University Hospital of Strasbourg, 67000 Strasbourg, France; 5 INSERM (French National Institute of Health and Medical Research), UMR 1260, Regenerative Nanomedicine (RNM), FMTS (Fédération de Médecine Translationnelle de Strasbourg), University of Strasbourg, 67000 Strasbourg, France

**Keywords:** hemodynamics, hemorrhagic shock, kidney, norepinephrine, renal hemodynamics, renal perfusion, vasopressin, vasopressor, ischemia/reperfusion

## Abstract

During hemorrhagic shock, blood loss causes a fall in blood pressure, decreases cardiac output, and, consequently, O_2_ transport. The current guidelines recommend the administration of vasopressors in addition to fluids to maintain arterial pressure when life-threatening hypotension occurs in order to prevent the risk of organ failure, especially acute kidney injury. However, different vasopressors exert variable effects on the kidney, depending on the nature and dose of the substance chosen as follows: Norepinephrine increases mean arterial pressure both via its α-1-mediated vasoconstriction leading to increased systemic vascular resistance and its β1-related increase in cardiac output. Vasopressin, through activation of V1-a receptors, induces vasoconstriction, thus increasing mean arterial pressure. In addition, these vasopressors have the following different effects on renal hemodynamics: Norepinephrine constricts both the afferent and efferent arterioles, whereas vasopressin exerts its vasoconstrictor properties mainly on the efferent arteriole. Therefore, this narrative review discusses the current knowledge of the renal hemodynamic effects of norepinephrine and vasopressin during hemorrhagic shock.

## 1. Introduction

Hemorrhagic shock may cause arterial hypotension and, consecutively, acute circulatory failure. Together with the control of the source of bleeding, fluid resuscitation and transfusion of blood products are recommended by the current guidelines [1]. Norepinephrine is referred to as the drug of first choice if vasopressors are additionally required to maintain adequate perfusion pressure [1]. However, the use of vasopressors per se is still a matter of debate, especially due to the risk of excessive vasoconstriction. In addition, the respective effect on the kidney of the vasopressor of the molecule chosen remains an open question. Therefore, this review discusses the pathophysiological rationale for the administration of the two most frequently used vasopressors during hemorrhagic shock, i.e., norepinephrine and vasopressin, with a special focus on the kidney.

## 2. Pathophysiology of Hemorrhagic Shock: Why Could We Need Vasopressor?

During hemorrhagic shock, blood loss causes the fall of blood pressure, decreases cardiac output, and, consequently, O_2_ transport [2], in turn leading to activation of the sympathetic system, which comprises arterial and venous vasoconstriction. Via this “compensatory phase” during the early period of hemorrhagic shock, the body aims at restoring macro-circulatory perfusion [3]. However, beyond a certain amount of blood loss, a sympatho-inhibitory response with hypo-responsiveness to vasopressor occurs, resulting in a vasoplegic state with arterial hypotension and, ultimately, potentially cardiac arrest [4]. Under these conditions, the administration of vasopressors appears to be a sensible approach.

In the second phase of hemorrhagic shock, once control of bleeding has been obtained, the patient may develop a sepsis-like response induced by the ischemia/reperfusion (I/R) sequence comprising oxidative and nitrosative stresses [5] as well as the systemic release of cytokines [6]. In addition, the use of analgesic and sedative drugs, which is mandatory for the management of patients with hemorrhagic shock, may further impair the vasoconstrictor response [7]. Finally, vasopressors, in particular norepinephrine, may help to stabilize hemodynamics as a result of splanchnic veno-constriction and the consecutive shift of blood volume into the central circulation caused by the increased pressure in capacitance vessels [1]. Therefore, the administration of vasopressors seems to be useful as an adjunct measure to restore vasomotor tone in both the early resuscitation as well as the post-reperfusion phase of the management of hemorrhagic shock [1,2,4].

## 3. Hemorrhagic Shock and Acute Kidney Injury

**Figure 1:**
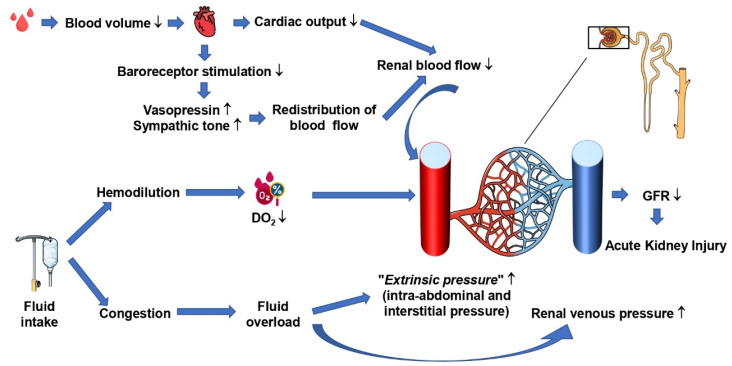
Physiopathology of acute kidney injury in patients with hemorrhagic shock. DO_2_: oxygen delivery; GFR: glomerular filtration rate.

During hemorrhagic shock, several mechanisms may induce acute kidney injury (AKI): (1) The fall of MAP and the consecutive decrease in CO are associated with reduced renal blood flow (RBF), O_2_ delivery, and microcirculatory perfusion [8,9,10] (Figure 1). 

(2) Through the hormonal activity, i.e., the above-mentioned activation of the sympathetic system, redistribution of blood to the “vital*”* organs, heart, and brain further reduce renal blood flow. 

(3) Within the renal parenchyma, RBF is redistributed at the expense of the renal cortex and outer medulla [11,12]. Since the microvascular O_2_ partial pressure (P_μv_O_2_) declines much earlier in the kidney than in other organs [13], AKI with glomerular and tubular injuries may occur [11]. In the absence of severe hypotension, the loss of hemoglobin associated with fluid resuscitation-induced hemodilution may decrease renal O_2_ supply with redistribution of P_μv_O_2_ away from the cortex and outer medullar [14], ultimately leading to impaired renal function [15,16] (Figure 1). During normovolemic hemodilution, despite preserved or even increased total RBF, cortex, and medulla P_μv_O_2_ drops immediately, and renal O_2_ consumption (VO_2_R) becomes dependent on renal O_2_ delivery (DO_2_R) [14]. Both Legrand et al. and Ergin et al. reported in rats that fluid resuscitation during hemorrhagic shock alone without additional vasopressor administration did not allow for restoring renal P_μv_O_2_ [8,9]. In addition, the re-transfusion of shed blood after canine hemorrhagic shock failed to restore VO_2_R and lactate uptake despite the increased renal P_μv_O_2_ [10]. 

While hemorrhagic shock situation per se may be responsible for the development of AKI, resuscitation therapies may also contribute to kidney injury as follows: restoration of blood flow due to the resuscitation procedure may induce renal I/R-injury as a result of oxidative and nitrosative stress [17]. Moreover, excessive fluid administration may cause congestive renal edema that decreases the glomerular filtration rate (GFR). Finally, as mentioned above, fluid administration may lead to hemodilution and thereby decrease O_2_ supply.

## 4. Renal Hemodynamics and Autoregulation

Within the normal MAP range, RBF and GFR are auto-regulated as follows: in fact, in conscious mammals, RBF and GFR remained unchanged above the MAP thresholds of 65 and 80 mmHg, respectively [18,19,20]. RBF autoregulation is based on the following two mechanisms: the myogenic response and the tubuloglomerular feedback.

The myogenic response is related to vascular smooth muscle cells’ contraction in response to stretching force [21]. In the kidney, an increase in arterial pressure leads to vasoconstriction of the renal afferent arteries. This mechanism appears to be protective against a rise in the glomerular capillary pressure, allowing it to maintain the glomerular flow unchanged [22]. 

The tubuloglomerular feedback leads to vasoconstriction of the renal afferent arteries in response to an increase in sodium chloride concentration in the macula densa in the early distal tubule [23]. An increase in sodium, chloride, and osmolarity concentration in the intra-distal tubular fluid leads to the activation of chemoreceptors in the macula densa [23]. This mechanism induces vasoconstriction of renal afferent arterioles in order to decrease the glomerular perfusion flow. As sodium reabsorption from the ascending part of the loop of Henle was an active and limited process, conversely to the passive diffusion of water out the descending loop of Henle, the concentration of sodium chloride reaching the macula densa was dependent on the rate of tubular flow as follows: the higher the renal tubular flow rate, the higher the distal tubular NaCl concentration. Therefore, the vasoconstriction of the renal arterial afferent led to a decrease in the RBF and a decrease in sodium chloride concentration in the distal tubular fluid. 

Therefore, a decrease in MAP will decrease tubular flow through a decreased glomerular filtration [23]. This will induce a decreased sodium chloride concentration at the macula densa, responsible for afferent arteriolar vasodilatation, providing restoration of RBF.

In this way, the nature and amount of chloride among resuscitation fluids became the object of many controversies in the setting of critically ill patients. No trial found improved mortality nor renal outcomes with the use of balanced fluids when compared to sodium chloride 0.9% [24].

However, below these critical thresholds of mean renal artery pressure, both RBF and GFR decreased and became MAP dependent. 

## 5. Rationale for the Use of Norepinephrine in Patients with Hemorrhagic Shock

Norepinephrine increases systemic vascular resistance through α-1 receptor activation. Furthermore, through β-1 activation, norepinephrine increases CO [25], and both effects together mediate an increase in MAP. Equivocal data are available on the renal hemodynamic effects of norepinephrine in healthy animals, in as much as increased [26], unchanged [27,28,29], or decreased [30,31] RBF has been reported. These different RBF responses have been referred to different effects on CO and/or to variable basal sympathetic tone.

In a model of a hemorrhagic pig, RBF was preserved between the range of 60 and 100 mmHg of MAP [32]. During hemorrhagic shock, the autoregulation mechanism may be impaired, and hence, RBF may become dependent on MAP [33,34]. To illustrate autoregulation failure, Vatner et al. induced moderate hypotensive controlled hemorrhage in dogs with a moderate MAP decreased, RBF remained unchanged, and renal arteries resistance decreased. Then, a further bleeding was induced, leading to a severe hemorrhage with a marked MAP decreased, RBF decreased, and renal resistance increased. These results suggested a loss of RBF autoregulation mechanism in the most severe hemorrhage [35]. In addition to the loss of autoregulation during severe hemorrhagic shock, Adams et al. reported that compared to control, kidney challenge with ischemia-reperfusion stress completely lost the autoregulation mechanism [36]. 

In healthy humans, norepinephrine infusion has been reported to reduce RBF [37,38,39,40,41] as a result of increased afferent and efferent glomerular arteriolar resistances [37]. Interestingly, vasoconstriction was more pronounced in efferent glomerular arteries, fostering the maintenance of the GFR [41,42]. In a situation with well-maintained autoregulation, norepinephrine increases renal vascular resistance through an α-receptor-mediated, direct vasoconstriction of both afferent and efferent renal arteries. The increase in glomerular capillary pressure leads to vasoconstriction of the afferent arteriole through the autoregulation phenomenon [43,44].

To summarize, the severity and ischemia-reperfusion stress during hemorrhagic shock both contribute to the loss of kidney autoregulation. Therefore, these results suggest a rationale for the use of norepinephrine to target MAP within physiological ranges in hemorrhagic shock states in order to maintain glomerular perfusion pressure. 

## 6. Renal Hemodynamic Effects of Norepinephrine during Hemorrhagic Shock 

As mentioned above, during hemorrhagic shock, norepinephrine induces veno-constriction, which may help to mobilize the unstressed blood venous compartment in order to increase the circulating blood volume [1,39]. Various experimental studies are available on the effects of norepinephrine on the kidney during hemorrhagic shock (for details, see Table 1).

In a dog model of hemorrhagic shock, norepinephrine-related titration of MAP values above 100 mmHg was associated and did not allow for restoring renal P_μv_O_2_ to pre-shock levels [47]. However, the MAP achieved was 110 mmHg, thus possibly causing excessive vasoconstriction. Moreover, in this experiment, shed blood was not re-transfused, suggesting a further decrease in renal DO_2_.

In rats undergoing hemorrhagic shock with MAP ~30 mmHg over 60 min, resuscitation with fluid resuscitation alone (i.e., re-transfusion of shed blood and Ringer’s lactate) was compared with a pre-established limited fluid volume (i.e., a bolus totalizing 40% of the blood volume initially withdrawn) plus norepinephrine both aiming at a MAP target of 50–55 mmHg. At day 1 or 3 post-shock, neither renal function nor markers of renal tissue injury showed any inter-group difference [45]. Moreover, in particular, this study confirmed the fluid-sparing effect of vasopressor administration during hemorrhagic shock demonstrated by others [49].

In a pig model of combined hemorrhagic shock (25–30 mL/kg of blood loss over 30 min) and blunt chest trauma, Prunet et al. reported lower urine output in the group resuscitated to a MAP of 70 mmHg with combined norepinephrine and fluids when compared to fluids alone [48]. However, it should be noted that CO and pulmonary artery occlusion pressure were significantly lower and stroke volume variation higher in the group treated with norepinephrine and fluid, as compared to the group treated with fluid only, suggesting more severe hypovolemia in that group.

In another model of porcine hemorrhagic shock, resuscitation to a systolic arterial pressure target of 80–90 mmHg with norepinephrine in combination with fluid administration restored kidney microcirculation and oxygenation, as well as renal function in a manner comparable to fluid resuscitation alone. However, at 48 h post-shock, additional norepinephrine administration led to a fluid volume-sparing effect with less hemodilution and, subsequently, an attenuated drop of hemoglobin concentration, as compared with fluid-alone resuscitation [46]. 

In summary, norepinephrine, in combination with fluids, probably does not alter the renal microcirculation and function during resuscitation hemorrhagic shock and allows the sparing fluids [49]. However, the association between excessive fluid overload and acute kidney injury [50] is well established as follows: accumulation of fluid and the consecutively increased renal venous and interstitial pressure will result in a reduced transrenal pressure gradient for RBF (Figure 1). Nevertheless, albeit beneficial effects of norepinephrine were reported in experimental models of hemorrhagic shock achieved by controlled bleeding. This situation is not comparable to the situation of major trauma with its inherent inflammatory responses, rhabdomyolysis, and potentially abdominal compartment that may further threaten kidney function. In addition, for obvious ethical reasons, in these experimental models, animals were under general anesthesia, and any benefit of infusing norepinephrine may have been the result of counteracting the anesthesia-related decrease in sympathetic tone. 

## 7. Rationale for the Use of Vasopressin in Patients with Hemorrhagic Shock

Vasopressin is synthesized in the hypothalamus and stored in the post-pituitary gland. Vasopressin secretion is regulated by plasma osmolarity as well as by blood volume and pressure. Vasopressin has well-known specific renal effects through the activation of V2 receptors located on the basolateral surface of renal tubular cells in collecting ducts. There, vasopressin induces aquaporine-2 recruitment, leading to increased permeability of the epithelial membrane to water and, consecutively, allowing water reabsorption [51]. In addition to V2 receptors, vasopressin also binds to V1a receptors, the stimulation of which induces vascular smooth cell contraction and, consequently, vasoconstriction [51]. Interestingly, V1a receptors distribution is heterogeneous in renal circulation. As a result, the vasoconstrictive properties of vasopressin infused at low doses has a predominant effect on the renal efferent arterioles, while their negligible effects are on the renal afferent arterioles. Apart from V1a receptors distribution, this variable effect on renal arterioles vasomotor tone appears to be related to a local phenomenon of nitrogen monoxide release [52,53]. Through the increase in efferent vasoconstriction, theoretically, glomerular renal perfusion pressure rises, and, consequently, also GFR. In fact, this rationale is consistent with clinical data that, outside the context of hemorrhagic shock, reports higher diuresis, higher creatinine clearance [54,55,56], and reduced need for renal replacement therapy [57] in patients receiving vasopressin administration. Moreover, when blood loss is severe, the initial activation of the sympathetic system to maintain MAP is no longer sufficient, with abnormal vascular bed reactions mediated by nitric oxide-dependent mechanisms that reduce the response to endogenous and exogenous norepinephrine [58]. Furthermore, a rapid fall in the levels of the circulating arginine-vasopressin peptide during hemorrhagic shock was reported [59]. Both phenomena theoretically support vasopressin administration during the management of the hemorrhagic shock.

## 8. Renal Hemodynamic Effects of Vasopressin during Hemorrhagic Shock 

Various experimental studies are available on the renal effects of vasopressin on the kidney during hemorrhagic shock (for details, see Table 2). In anesthetized and hypovolemic animals, vasopressin was shown to not only increase MAP but also CO and, consecutively, RBF. In fact, such macro-hemodynamic effects were reported by Voelcker et al. in swine undergoing uncontrolled, near-fatal hemorrhagic shock (MAP < 20 mmHg, shock-related > 30% fall of heart rate): not only did vasopressin administration improve survival when compared to crystalloid fluid resuscitation alone [60,61], but also compared favorably with epinephrine after liver laceration-induced hemorrhage [62] and even after hemorrhage-induced cardiac arrest [62]. So far, no studies are available evaluating the effect of vasopressin on renal microcirculation and oxygenation during and after hemorrhagic shock; nevertheless, in rats undergoing decompensated hemorrhagic shock with MAP ~40 mmHg and resuscitated with lactated Ringer’s over 60 min, combining fluids with vasopressin renal tissue mitochondrial respiratory activity, attenuated formation of reactive oxygen species and thereby lipid peroxidation-mirrored oxidative damage and histological injury at 18 h post-shock [63].

These experimental findings prompted the authors’ group to perform the so far single clinical study that investigated the impact of vasopressin on renal function in patients with hemorrhagic shock. In a randomized, double-blind, placebo-controlled trial in a total of 100 patients with trauma, who had received at least 6 units of blood products, the authors tested the hypothesis of whether vasopressin (bolus of 4 U, thereafter ≤0.04 U/min) vs. other vasopressors and titrated to target a MAP ~65 mmHg would reduce the total volume of blood product transfused. Patients who received AVP indeed required significantly fewer blood products, while none of the other secondary end points (i.e., total fluid balance (*p* = 0.10), vasopressor requirements, secondary complications, and mortality at day 30) showed any significant intergroup difference. Of note, albeit not significant either (*p* = 0.19), AKI was less frequent in the AVP-treated patients (*n* = 8, 16%) when compared to the control group (*n* = 14, 27%) [65]. This result is interesting as blood product administration may independently be associated with the following adverse events: venous thromboembolism, multiple-organ failure, and death [66,67]. 

## 9. Potential Adverse Effect of Vasopressor during Hemorrhagic Shock

During hemorrhagic shock, arterial vascular resistances are increased, and cardiac output is low as well. The use of norepinephrine alone may theoretically worsen the hemodynamics, may induce excessive vasoconstriction, and may further induce ischemic injuries. Experimental studies, due to their short-term assessment, do not allow to answer this concern, and in a clinical setting, there is no data on patients with hemorrhagic shock treated with vasopressor alone.

Furthermore, there is a theoretical risk of lowered cardiac output through higher cardiac afterload induced by arterial vasoconstriction with norepinephrine use. This was not reported in the literature. This may be explained by the beta1 effect of norepinephrine. 

## 10. Conclusions

The European guidelines recommend the administration of vasopressors in addition to fluids to maintain the target arterial pressure in the presence of life-threatening hypotension [1]. These recommendations are based on studies where patients with hemorrhagic shock resuscitated with restricted volume and permissive hypotension had either improved survival [68,69,70] or at least unchanged mortality [71,72] when compared to patients resuscitated with a non-restrictive fluid strategy. In addition, aggressive volume administration has been shown to aggravate the incidence of secondary abdominal compartment syndrome [73], coagulopathy [74], and multiple organ failure [75] and, thereby, decrease the likelihood of survival [75,76,77,78]. 

To date, no randomized studies compared the outcome of patients with hemorrhagic shock resuscitated with fluids alone vs. fluids with vasopressor. Moreover, no randomized study compared the choice of vasopressor used in this situation. In this mini-review, we highlighted the importance of this unanswered question and the utmost importance of renal hemodynamic effects of vasopressors in the setting of hemorrhagic shock. Nevertheless, according to the current knowledge, we might suggest that vasopressor treatment could be used during hemorrhagic shock in association with fluid therapy. Norepinephrine administration appears to be a safe approach, as it does not threaten kidney function and allows a fluid-sparing effect. To date, there is not enough data to evaluate and conclude the impact of vasopressin on kidney hemodynamics and/or function.

## Figures and Tables

**Table 1:** Norepinephrine and kidney during hemorrhagic shock. MAP: mean arterial pressure, SAP: systolic arterial pressure.

References	Species	Models of Hemorrhagic Shock	Arterial Pressure Target during Resuscitation	Intervention	Group Compare	Main Results	Limitations
Dunberry-Poissant et al. [45]	Anesthetized wistar rats	Blood exsanguination to target MAP 30 mmHg during 60 min, resuscitation after control of bleeding	55–60 mmHg of MAP target	Resuscitation with 40% of the shed blood withdrawn then used of norepinephrine	- Resuscitation with fluid only	- No difference between delta of creatininemia (baseline—end of reperfusion) according to the 2 groups - Similar increases of proteins markers of kidney injury - Less fluid was needed for resuscitation	- Model of haemorrhagic shock without trauma
Libert et al. [46]	Anesthetized pigs	Blood exsanguination to MAP target 30 mmhg–35 mmHg during 90 min, resuscitation after stop of exsanguination	80–85 mmHg of SAP target	Resuscitation with norepinephrine and fluid	- Resuscitation with fluid only - Placebo	- Combination of norepinephrine and fluid restored renal microcirculation, oxygenation, and renal function as fluid alone. - Higher renal histological damage in resuscitation with fluid only - Less fluid was needed for resuscitation	- Model of haemorrhagic shock without trauma
Murakawa et al. [47]	Anesthetized dogs	Blood exsanguination to MAP target 50 mmHg during 60 min,	>100 mmHg of MAP target for 90 min	Resuscitation with norepinephrine	- Resuscitation with dopamine - Resuscitation with epinephrine - Placebo	- No increase in renal tissue oxygen tension during resuscitation with norepinephrine	- Physiological study with no randomization - No statistical analysis - Excessive MAP target
Prunet et al. [48]	Anesthetized pigs	Chest trauma and blood exsanguination to reach MAP target of 50 mmHg during 90 min	MAP to 70 mmHg	Resuscitation with limited fluid and norepinephrine	- Resuscitation with fluid without norepinephrine - Placebo	- Lower urine output in norepinephrine group	Lower cardiac output in group with use of norepinephrine

**Table 2:** Vasopressin and kidney during hemorrhagic shock. MAP: mean arterial pressure.

References	Species	Models of Hemorrhagic Shock	Arterial Pressure Target during Resuscitation	Intervention	Group Compare	Main Results	Limitations
Voelckel et al. [62]	Anesthetized pigs	Models of very severe haemorrhagic shock: Dissection of the right liver lobe allowing blood loss, to reach MAP target < 30 mmHg (near fatal hypotension)	Increase MAP without a specific target.	Resuscitation with vasopressin during uncontrolled shock	- Flush of epinephrine - Placebo	In vasopressin group, renal artery blood flow was restored and remains higher than epinephrine or placebo groups	No information on the effect on renal function
Voelckel et al. [64]	Anesthetized pigs	Models of very severe haemorrhagic shock: Blood exsanguination and ventricular fibrillation was induced with single administration of alternating current.	Return of spontaneous circulation with a MAP ≥ baseline value before exsanguination.	Injection of vasopressin after 4 min of untreated ventricular fibrillation and 4 min of cardiopulmonary resuscitation	- Flush of epinephrine - Placebo	In vasopressin group, renal artery blood flow was restored and remains higher than epinephrine or placebo groups	No information on the effect on renal function
Sims et al. [65]	Humans	Trauma patients who received at least 6 units of blood products	MAP target ≥ 65 mmHg for 48 h	Randomized study: use of Vasopressin (bolus of 4 U then 0.04 U/min) ± norepinephrine to target ≥ 65 mmHg of MAP	- Placebo	- Higher urine output in vasopressin group - No difference in renal function - Lower blood products requirement in vasopressin group	- Low number of patients (100)

## Data Availability

No new data were created or analysed in this article.

## Abbreviations

AKI	Acute kidney injury
CO	Cardiac output
DO_2_	Oxygen delivery
DO_2_R	Renal oxygen delivery
GFR	Glomerular filtration rate
I/R	Ischemia/Reperfusion
MAP	Mean arterial pressure
P_μv_O_2_	Microvascular O_2_ partial pressure
RBF	Renal blood flow
SAP	Systolic arterial pressure
VO_2_R	Renal oxygen consumption
